# Who counts as diverse? The strategic broadening and narrowing of diversity

**DOI:** 10.3389/fpsyg.2024.1297846

**Published:** 2024-02-06

**Authors:** Junming Zhang, Teri A. Kirby

**Affiliations:** Department of Psychological Sciences, Purdue University, West Lafayette, IN, United States

**Keywords:** diversity, social identity, inclusion, intergroup relation, diversity definition, colorblind ideology

## Abstract

**Introduction:**

A large majority of US organizations profess a commitment to diversity, but their definitions of diversity can vary greatly. While previous research demonstrates a shift in diversity definitions to include fewer protected demographic groups and more non-demographic characteristics, the present research examines whether this shift might be a motivated process among dominant group members related to anti-egalitarian and colorblind belief systems.

**Methods:**

Using quantitative and qualitative methods, we explored potential underlying ideologies that may be associated with White Americans’ shifting definitions of diversity. White Americans (*N* = 498) were asked how they define diversity, as well as who should be included in a range of diversity initiatives.

**Results:**

White participants’ higher anti-egalitarian belief was associated with stronger colorblind ideology endorsement, which was then associated with shifting their definition of diversity to include fewer disadvantaged demographic groups, more advantaged demographic groups, and non-demographic groups, as well as employing a colorblind inclusion rhetoric.

**Discussion:**

Instead of only “broadening” diversity to include more characteristics than diversity’s original focus, White Americans higher in anti-egalitarian and colorblind motives exhibited a simultaneous “narrowing” of diversity to include fewer protected demographic characteristics. Taken together, these findings have implications for dominant group members’ definition of diversity and the subtle ways in which colorblind ideology may be enacted.

## Introduction

A large majority of organizations in the United States (U.S.) profess a commitment to diversity (Kirby et al., 2023). How people and organizations define diversity can vary greatly, however (Howard et al., 2021; Kirby et al., 2023). While diversity and diversity initiatives originally served to increase the representation of oppressed and marginalized group members, organizational definitions of diversity have expanded to include individual traits (e.g., personality, ideology) that are not protected by law (Edelman et al., 2001). For example, a worldwide employment website describes workplace diversity as “the individual characteristics employees have that make them unique,” including “employees’ life experiences, how they solve issues, and socioeconomic status” (Indeed, n.d.). This pattern is also reflected in the diversity statements of the top 250 Fortune companies, where references to non-demographic characteristics increased between 2014 and 2020 (Kirby et al., 2023). This new expanded definition of diversity appears to include virtually *everyone* and insinuates a shift away from diversity’s focus on protected and marginalized identities.

In the present research, we aim to gather preliminary evidence for *why* this shift may be occurring. In line with previous research demonstrating the role of individuals’ intergroup beliefs in their definitions of diversity (Unzueta and Binning, 2012; Unzueta et al., 2012; Danbold and Unzueta, 2020), we argue that shifting definitions of diversity may be a motivated process among dominant group members. In particular, we aim to understand how White Americans’ anti-egalitarian belief is associated with colorblind endorsement and therefore shifting definitions of diversity with less focus on disadvantaged demographic groups.

### Anti-egalitarian belief and diversity construal

Anti-egalitarian belief reflects the extent to which people support social hierarchy and inequality. Individuals high in anti-egalitarian belief (i.e., anti-egalitarian individuals) prefer hierarchical group orientations and dominance over low-status groups, while individuals low in anti-egalitarian belief (i.e., egalitarian individuals) support egalitarianism within and between groups (Sidanius and Pratto, 1999; Ho et al., 2015). Among White Americans, higher endorsement of anti-egalitarian belief is associated more prejudice against ethnic outgroups (Kteily et al., 2011).

Anti-egalitarian belief may have a notable impact on how dominant group individuals understand and perceive diversity. Previous research has suggested that people construe the meaning of diversity in ways that serve their anti-egalitarian motives (Unzueta et al., 2012). In particular, anti-egalitarian participants “broaden” their definitions of diversity by judging an organization as more diverse if it is high in occupational heterogeneity (i.e., more even distribution of workforce types), even if it is low in racial heterogeneity−they then use this to legitimize their opposition to affirmative-action policies (Unzueta et al., 2012). Additionally, compared to minoritized group members, dominant group members consider organizations to be “diverse” at lower numerical representations of minoritized group members, which is driven by a desire to maintain their standing in the social hierarchy (Danbold and Unzueta, 2020).

Similar to individuals’ construal of diversity, the concept of “discrimination” can also be defined narrowly or broadly, depending on individuals’ definitions of discrimination (Greenland et al., 2022). Specifically, dominant group members strategically employ the broad and narrow definitional boundary of discrimination motivated by their ingroup-serving and hierarchy-maintaining motivations (West et al., 2021, 2022). For example, when asked what counts as “discrimination”, White male participants included a wider range of behaviors under the label “discrimination” when identifying discrimination against their ingroup; however, they included a narrower range of behaviors when identifying discrimination against their outgroup (West et al., 2022). Notably, these patterns only appear for White men with high levels of anti-egalitarian belief, suggesting their tendency to construe “discrimination” in line with their belief systems.

Consistent with these findings of motivated construal of diversity and discrimination, we propose that anti-egalitarian belief will affect dominant group members’ overall conception of *who* counts as diverse. We expect dominant group members’ anti-egalitarian belief to be associated with more broadening of diversity to include more non-demographic groups, as well as advantaged demographic groups, as a means to include themselves in diversity. Simultaneously, anti-egalitarian belief will be associated with narrower definitions of diversity to include fewer disadvantaged demographic groups, consistent with their motives of maintaining their dominant social statuses.

### Colorblind racial ideology

Why might anti-egalitarian belief be associated with these shifting definitions of diversity? Colorblind racial ideology, or colorblindness, is one underlying ideology that may result in a desire to obscure a focus on protected characteristics and the realities of discrimination. Specifically, colorblindness is an ideology that downplays racial/ethnic identities to focus on individual uniqueness or commonalities with others (Gündemir and Kirby, 2022). Although colorblind ideology could theoretically orient individuals toward equality and intergroup harmony by advocating for intergroup equality and non-discrimination, components of colorblindness can instead serve hierarchy-enhancing ends (Neville et al., 2013; Whitley et al., 2022). For example, endorsing colorblind ideology is associated with higher anti-Black racism, more beliefs that justify societal inequality, and higher ingroup favoritism (Whitley et al., 2022; Yi et al., 2023). Moreover, exposing dominant group members to messages endorsing colorblindness leads to higher levels of explicit and implicit racial bias (Richeson and Nussbaum, 2004; Holoien and Shelton, 2012).

Colorblindness is also theorized as a form of “new racism” that White Americans uphold to ignore race-based inequalities and injustices and to look another way (Bonilla-Silva, 2003, 2015). Endorsing colorblind ideology and utilizing colorblind rhetoric allows White Americans to justify and rationalize contemporary racial inequality, minimize prevalent racial prejudice and discrimination, and deny their existing privilege (Bonilla-Silva, 2015). Compared to racial minority students, White college student participants more often exhibit colorblind racial ideology by adopting an “everyone is diverse and unique” mindset (Dingel and Sage, 2020). Some participants exhibited a “laundry-list approach” when describing diversity, where they classify a wide variety of traits as relevant to diversity—many of which are irrelevant to protected demographic identities (Dingel and Sage, 2020). This “laundry-list approach” exhibits entrenched colorblind thinking in its approach of including everyone in diversity; it also demonstrates how an “all-inclusive” definition can obscure systematic inequality (Dingel and Sage, 2020). Therefore, colorblind ideologies might be an appealing strategy employed by individuals who are more anti-egalitarian to obfuscate systematic inequality. We use the term “colorblind-inclusion” to refer to an ideology that includes *everyone* in diversity (i.e., the ideology is inclusive by definition, but enacts a form of colorblindness).

Accordingly, we expect anti-egalitarian belief to be associated with endorsement of colorblindness, and therefore White Americans adopting a “colorblind inclusion” mindset and considering non-protected demographic groups and advantaged demographic groups as part of their conceptualization of diversity. One possibility is that colorblind-inclusion will manifest as including a range of groups as part of their diversity definitions, including protected-demographic groups. However, because colorblindness downplays race-based inequalities and historical oppression, it could also be associated with White participants being less likely to include disadvantaged demographic groups in diversity.

### Present research

Past research has demonstrated that definitions of diversity are shifting to include more non-demographic groups (Edelman et al., 2001; Kirby et al., 2023). The present research aims to understand the underlying ideologies that may be associated with this process among White Americans. Specifically, we predict that anti-egalitarian attitudes will be associated with stronger colorblind endorsement, which will be associated with including fewer disadvantaged demographic groups (e.g., racial minorities), more non-demographic groups (e.g., mathematical thinkers), and more advantaged demographic groups (e.g., White people) in their conceptions of diversity^1^.

## Materials and methods

### Participants

We recruited 549 White undergraduate participants from the participant pool at a public Midwestern University in the U.S. We excluded 19 participants who were under the age of 18, 26 who identified as a race other than White, and 6 who failed the manipulation check, leaving a final sample of 498 (age *M* = 18.63, *SD* = 0.96). Of these, 320 identified as women, 174 identified as men, and 4 identified as non-binary or another identity. The majority (78%) of participants indicated U.S. American as their nationality.

As pre-registered^2^ we needed to recruit 352 participants to obtain *d* = 0.3 according to the t-test function for two independent groups in GPower (Faul et al., 2009). To account for possible participant exclusions, we aimed to collect data from 375 participants. Given our obtained sample size, a sensitivity analysis using GPower 3.1 suggested that we could detect an effect size as small as η^2^ = 0.02 with 80% statistical power at an alpha level of 0.05.

### Procedure

Participants were brought into the lab by research assistants and completed the survey on lab computers. In a 2-level design, they were randomly assigned to either read about changing demographics at their university, where racial minorities will become the majority of the student body, or a control article about geographic mobility after graduation (adapted from Craig and Richeson, 2014). While the original manipulation describes either shifting racial demographics or shifting geographic mobility of United States citizens (Craig and Richeson, 2014), our adaptation discusses shifts in the university student body. After reading the manipulation article, they completed the dependent measures in the order described below, as well as manipulation checks and demographics.

### Measures

#### Count measure of diversity definition

To determine participants’ definitions of diversity, they decided which identities should be included in four campus diversity initiatives (mentoring, college application outreach program, having a designated space on campus, and extra resources) and also directly responded about who they included in their definition of diversity. They read a list of 21 identities that included 9 disadvantaged demographic groups (e.g., black people; α = 0.98), 9 non-demographic groups (e.g., mathematical thinkers; α = 0.98), and 3 advantaged demographic groups (e.g., white people; α = 0.93) and responded on a scale from 1 (definitely do not include) to 6 (definitely include). Because the anchors had no midpoint, the measure served as a forced choice inclusion or exclusion measure. See Table 1 for specific groups included in each category.

**TABLE 1:** Quantitative definition categories.

Category name	Groups included in the category
Disadvantaged Demographic Groups	Gay or lesbian people, transgender people, women, Black people, Muslim people, Asian Americans, Native Americans, neurodivergent people (e.g., people with autism), and people with physical disabilities
Non-demographic Groups	introverts, free spirited thinkers, people who are night owls, mathematical thinkers, visual learners, left-handed people, passive communicators, tactile learners, and deductive problem solvers
Advantaged (or Neutral) Demographic Groups	White people, Christian people, and conservative people

We pre-registered that we would first create a mean of participants’ overall desire to include the three different categories of groups in the diversity initiatives and definition as our primary dependent measure (our pre-registered hypothesis). We pre-registered we would then create another measure where we dichotomize participants’ answers in a binary variable and average the total number of groups they included for each category. We collapsed across conditions and shifted our focus to understand variables that might be associated with diversity definition shift. Thus, we chose to have the latter variable (the dichotomized measure) as our primary measure of diversity definition shift since it conceptually aligns with our research questions. Specifically, the dichotomous measure directly denotes participants’ conception of “who” counts as diverse. We report the mediation results for the first diversity definition measure in the Supplementary material, but it fully replicates the findings reported in the main text for the count measure.

We first dichotomized participants’ answers into a binary variable, where responses ranging from 1 to 3 (definitely do not include to maybe do not include) were recoded as 0 (i.e., exclude) and responses ranging from 4 to 6 (definitely include to maybe include) were recoded as 1 (i.e., include). Next, we summed the number of groups of each category participants included within the five initiative types. Finally, we created a mean across the initiative types go give a single mean sum for each identity type: disadvantaged demographic groups (*M* = 8.29, *SD* = 1.17), non-demographic groups, *M* = 5.60, *SD* = 2.68), and advantaged demographic groups (*M* = 2.19, *SD* = 0.82).

#### Open-ended definition of diversity

To assess participant’s definition of diversity, they answered the question “What factors should determine if a group should be included in a diversity initiative (e.g., who should be included in diversity efforts?)? Do different groups matter in different ways? Why do you feel that way?” with an open-ended response. Their responses were then coded by two research assistants. See Table 2 for content coding categories. Research assistants coded responses for whether participants discussed each of the categories with the following codes: −1 = Mentioned (should not be included), 0 = Not mentioned, 1 = Mentioned (should be included). Because mentioning that a group should be excluded was rare (*n* < 10), we recoded these values (−1) into 0, such that the variables were binary (1 = Group should be included, 0 = Group should be excluded or wasn’t mentioned). We also coded for colorblind inclusion rhetoric, where coders assessed whether participants’ responses suggested that everyone should be included in diversity, or that no particular groups should be prioritized over others.

**TABLE 2:** Content coding categories.

Category name	Definition of category
Specific disadvantaged demographic groups	Disadvantaged demographic groups that are protected by law from discrimination, such as ethnicity, race, gender, sex, sexual orientation, nationality (includes language, being from another place), religion, disability status, or age.
Non-specific disadvantaged demographic groups	Specific disadvantaged groups are not listed, but participant discusses groups that have experienced stigmatization in the past more generally (e.g., “minority groups,” “underrepresented groups”)
Non-demographic groups	Individual characteristics, such as personality, skills, abilities, perspectives, beliefs, talents, life experiences, background, working styles, work expertise, professional experience, or political views
Advantaged demographic groups	Advantaged demographic groups such as White people, men, Christians
Colorblind inclusion	Response suggests that everyone should be included or that no particular groups should be prioritized over others (e.g., “people from all different types of backgrounds should be included”)

After coding two practice rounds of 20 statements to refine the coding categories, research assistants coded the full set. When discrepancies arose, research assistants discussed until they agreed on how to code the response.

#### Anti-egalitarian beliefs

Participants indicated their agreement with eight items from the shortened Social Dominance Orientation scale (SDO_7(S)_; Ho et al., 2015; α = 0.80) measuring their anti-egalitarian beliefs on a 1 (*Strongly Disagree*) to 7 (*Strongly Agree*) scale (e.g., “An ideal society requires some groups to be on top and others to be on the bottom”). We averaged all items to form a measure where higher values corresponded to higher anti-egalitarian beliefs.

#### Colorblindness

We measured colorblindness with the Color Evasion subscale of the Multidimensional Assessment of Racial Colorblindness scale (Whitley et al., 2022; α = 0.92; e.g., “Talking about racial issues causes unnecessary tension”). We focused on the Color Evasion subscale because it reflects a desire to downplay the importance of race and ethnicity and instead highlight similarities (Whitley et al., 2022). Participants indicated their agreement with nine items on a 1 (*Strongly Disagree*) to 7 (*Strongly Agree*) scale. We averaged all items to form a measure where higher values corresponded to higher colorblind endorsement.

#### Political orientation

To assess participants’ political orientation, they answered two questions (“What is your political ideology with respect to social issues?” “What is your political ideology with respect to economic issues?”) on a 1 (*Extremely Liberal*) to 7 (*Extremely Conservative*) scale (α = 0.78). We averaged the two items to form a measure where higher values corresponded to more conservative political orientation.

## Results

### Analytic strategy

The demographic shift manipulation had an effect on one of three dependent measures, *p* < 0.001, *d* = 2.67. Because it was the opposite of our hypotheses and past findings (Craig and Richeson, 2014) and only emerged on one out of three measures, we believe it should be interpreted cautiously. Thus, we shifted our focus to exploratory analyses understanding potential variables that are associated with diversity definition shift (collapsed across experimental conditions).^3^ We report all original pre-registered analyses in the online Supplementary.

Specifically, we ran multiple regression analyses with colorblindness and anti-egalitarian beliefs as independent measures and the indices of diversity definition shifts as dependent measures. We used the PROCESS macro version 4.2 (Model 4, 10,000 bootstraps; Hayes, 2013) to test whether colorblindness mediated the relationship between anti-egalitarian belief beliefs and diversity definition shifts, operationalized as the inclusion of disadvantaged demographic groups, non-demographic groups, advantaged demographic groups, and the use of colorblind inclusion rhetoric. Since the qualitative dependent variables are binary variables, we utilized PROCESS macro’s function to run logistic regressions on the binary dependent variables.

To assess whether our proposed model held beyond the effects of political orientation, we ran all the above mediation analyses controlling for political orientation. We also examined political orientation as an alternative predictor variable (in place of anti-egalitarian belief) in the mediation model. We report the results in the online Supplementary.

Previous research has also demonstrated that anti-egalitarian belief moderates the association between colorblindness and outgroup attitudes, suggesting the possibility that anti-egalitarian belief moderates the association between colorblindness and diversity definition shift (Yogeeswaran et al., 2017). Because the results from the moderation model were unexpected and showed divergent patterns across dependent measures, we believe they should be interpreted cautiously until they are replicated. They are reported in full in the online Supplementary^4^.

### Preliminary analyses

Descriptive statistics and correlations between social dominance beliefs, colorblindness, and all diversity definition variables are reported in Table 3. In participants’ open-ended responses on diversity definition, 223 (44.8%) participants mentioned specific disadvantaged demographic groups, and 121 (24.3%) participants mentioned disadvantaged demographic groups in general ways (e.g., “minority groups”). Moreover, 55 (11%) participants mentioned non-demographic groups to be included in definition of diversity, and 17 (3.4%) mentioned advantaged demographic groups in their definition of diversity. Lastly, 159 (31.9%) participants used the colorblind-inclusion rhetoric, where they claimed that everyone should be included in diversity or that no particular group should be prioritized over others.

**TABLE 3:** Descriptive statistics and correlations for study variables.

Variable	*M*	*SD*	1	2	3	4	5	6	7	8	9	10	11
1. Social Dominance Orientation	2.49	0.92	—										
2. Colorblindness	3.55	1.51	0.49**	—									
3. Political Orientation	3.99	1.30	0.45**	0.61**	—								
4. Quantity of Disadvantaged Demographic Groups Included	8.29	1.17	−0.31**	−0.30*	−0.28**	—							
5. Quantity of Non-Demographic Groups Included	5.60	2.68	−0.00	0.13**	0.10*	0.35**	—						
6. Quantity of Advantaged Demographic Groups Included	2.19	0.82	0.09*	0.21**	0.26**	0.45**	0.70**	—					
	*n*	%											
7. Mention of Specific Disadvantaged Demographic Groups	223	44.8	−0.07	−0.09*	−0.11*	−0.01	−0.11*	−0.07	—				
8. Mention of Non-Specific Disadvantaged Demographic Groups	121	24.3	−0.05	−0.12**	−0.16**	0.02	−0.20**	−0.22**	−0.50**	—			
9. Mention of Non-demographic Groups	55	11.0	0.06	0.00	0.04	0.00	0.11*	0.09*	0.13**	−0.14**	—		
10. Mention of Advantaged Demographic Groups	17	3.4	−0.06	−0.05	−0.08	0.05	0.02	0.07	0.14**	−0.06	0.11*	—	
11. Use of Colorblind Inclusion Rhetoric	159	31.9	0.04	0.14**	0.13**	0.09*	0.33**	0.33**	−0.30**	−0.25**	−0.04	−0.03	—

*p < 0.05,

**p < 0.01.

Stronger social dominance orientation was associated with including fewer disadvantaged demographic characteristics, but was not consistently associated with inclusion of other characteristics (see Table 3). Stronger colorblindness was also associated with including fewer disadvantaged demographic characteristics, as well as *more* advantaged demographic and non-demographic characteristics—albeit more consistently for the quantitative than the qualitative open-ended coding measures.

### Main analyses

#### Quantitative diversity definition shift

Consistent with expectations, higher levels of social dominance orientation were associated with higher levels of colorblindness, *b* = 0.81, *SE* = 0.06, *p* < 0.001 (path *a*). Colorblindness, in turn, was significantly associated with including fewer disadvantaged demographic groups, *b* = −0.15, *SE* = 0.04, *p* < 0.001, more non-demographic groups, *b* = 0.30, *SE* = 0.09, *p* = 0.001, and more advantaged demographic groups, *b* = 0.11, *SE* = 0.03, *p* < 0.001, when controlling for social dominance orientation (path *b*).

**FIGURE 1:**
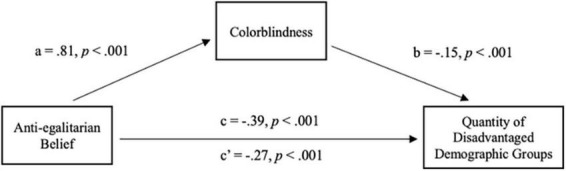
Regression coefficients for the relationship between Social Dominance Orientation and quantity of disadvantaged demographic groups included by participants as mediated by color evasion.

The mediation models showed significant indirect effects for disadvantaged demographic groups, non-demographic groups, and advantaged demographic groups. Specifically, social dominance orientation was associated with colorblind endorsement, which was associated with participants including fewer disadvantaged demographic groups, *b* = −0.12, *SE* = 0.04, 95% C.I. [−0.20, −0.05], more non-demographic groups, *b* = 0.24, *SE* = 0.08, 95% C.I. [0.09,0.39], and more advantaged demographic groups, *b* = 0.09, *SE* = 0.02, 95% C.I. [0.05,0.14] (see Figure 1 for one example mediation model and Table 4 for full mediation pathway results).

**TABLE 4:** Mediation pathway results for diversity definition shift variables.

	*b*	*SE*	*p*
**Model: SDO → Colorblindness → Quantity of Disadvantaged Demographic Groups**			
a (SDO → Colorblindness)	0.81	0.06	< 0.001
b(Colorblindness → Quantity of Groups)	−0.15	0.04	< 0.001
c(SDO → Quantity of Groups)	−0.39	0.05	< 0.001
c’ (Direct Effects)	−0.27	0.06	< 0.001
**Model: SDO → Colorblindness → Quantity of Non-demographic Groups**			
a (SDO → Colorblindness)	0.81	0.06	< 0.001
b(Colorblindness → Quantity of Groups)	0.30	0.09	0.001
c (SDO → Quantity of Groups)	−0.01	0.13	0.952
c’ (Direct Effects)	−0.25	0.15	0.093
**Model: SDO → Colorblindness → Quantity of Advantaged Demographic Groups**			
a (SDO → Colorblindness)	0.81	0.06	< 0.001
b(Colorblindness → Quantity of Groups)	0.11	0.03	< 0.001
c(SDO → Quantity of Groups)	0.08	0.04	0.035
c’ (Direct Effects)	−0.01	0.05	0.849
**Model: SDO → Colorblindness → Mention of Specific Disadvantaged Demographic Group**			
a (SDO → Color Evasion)	0.81	0.06	< 0.001
b(Colorblindness → Mention of Group)	−0.10	0.07	0.158
c’ (Direct Effects)	−0.07	0.11	0.529
**Model: SDO → Colorblindness → Mention of Non-specific Disadvantaged Demographic Group**			
a (SDO → Colorblindness)	0.81	0.06	< 0.001
b(Colorblindness → Mention of Group)	−0.20	0.08	0.018
c’ (Direct Effects)	0.02	0.13	0.894
**Model: SDO → Colorblindness → Mention of Non-demographic Group**			
a (SDO → Colorblindness)	0.81	0.06	< 0.001
b(Colorblindness → Mention of Group)	−0.08	0.11	0.476
c’ (Direct Effects)	0.27	0.18	0.124
**Model: SDO → Colorblindness → Mention of Advantaged Demographic Group**			
a (SDO → Colorblindness)	0.81	0.06	< 0.001
b(Colorblindness → Mention of Group)	−0.10	0.19	0.595
c’ (Direct Effects)	−0.30	0.34	0.367
**Model: SDO → Colorblindness → Use of Colorblind Inclusion Rhetoric**			
a (SDO → Colorblindness)	0.81	0.06	< 0.001
b(Colorblindness → Use of Rhetoric)	0.22	0.07	0.003
c’ (Direct Effects)	−0.08	0.12	0.486

*Note.* SDO = social dominance orientation

Because of the limitations of cross-sectional mediation analysis (see Fiedler et al., 2018), we also tested the reverse pathway (see Table 5). This pathway revealed significant indirect effects for the quantity of disadvantaged demographic groups, but not for the quantity of advantaged demographics groups. Although this suggests that this alternative model is possible, the other model has slightly more consistent results, and we consider our proposed pathway to be more theoretically plausible.

**TABLE 5:** Indirect effects from mediation models.

	Social Dominance Orientation→ Colorblindness → Dependent Variable			Colorblindness → Social Dominance Orientation → Dependent Variable		
Dependent Variable	*b*	*SE*	95% CI	*b*	*SE*	95% CI
Quantity of Disadvantaged Demographic Groups	−0.12	0.04	[−0.20, −0.05]	−0.08	0.02	[−0.14, −0.04]
Quantity of Non-demographic Groups	0.24	0.08	[0.09, 0.39]	−0.07	0.04	[−0.16, 0.01]
Quantity of Advantaged Demographic Groups	0.09	0.02	[0.05, 0.14]	−0.01	0.01	[−0.03, 0.02]
Mention of Specific Disadvantaged Demographic Group	−0.08	0.06	[−0.20, 0.03]	−0.02	0.04	[−0.09, 0.05]
Mention of Non-specific Disadvantaged Demographic Group	−0.16	0.07	[−0.31, −0.03]	0.01	0.04	[−0.07, 0.08]
Mention of Non-demographic Group	−0.06	0.10	[−0.27, 0.12]	0.08	0.06	[−0.02, 0.19]
Mention of Advantaged Demographic Group	−0.08	0.17	[−0.46, 0.23]	−0.09	0.10	[−0.31, 0.08]
Use of Colorblind Inclusion Rhetoric	0.18	0.06	[0.06, 0.31]	−0.03	0.04	[−0.10, 0.05]

#### Qualitative diversity definition shift

The direct effects for the qualitative diversity definition variables showed that colorblindness was negatively associated with participants mentioning disadvantaged demographic groups in non-specific ways, *b* = −0.20, *SE* = 0.08, *p* = 0.018, and positively associated with participants using the colorblind inclusion rhetoric, *b* = 0.22, *SE* = 0.07, *p* = 0.003, when controlling for social dominance orientation (path *b*). However, colorblindness was not significantly associated with participants mentioning specific disadvantaged demographic groups, *b* = −0.10, *SE* = 0.07, *p* = 0.158, mentioning non-demographic groups, *b* = −0.08, *SE* = 0.11, *p* = 0.476, and mentioning advantaged demographic groups, *b* = −0.10, *SE* = 0.19, *p* = 0.595.

Inconsistent with our quantitative measure, the mediation tests revealed that colorblindness did not mediate the association between social dominance orientation and participants’ mention of specific disadvantaged demographic groups, *b* = −0.08, *SE* = 0.06, 95% C.I. [−0.20,0.03], non-demographic groups, *b* = −0.06, *SE* = 0.10, 95% C.I. [−0.27,0.12], or advantaged demographic groups, *b* = −0.08, *SE* = 0.17, 95% C.I. [−0.46,0.23]. However, consistent with our quantitative measure, colorblindness significantly mediated the association between social dominance orientation and participants’ mention of non-specific disadvantaged demographic groups, *b* = −0.16, *SE* = 0.07, 95% C.I. [−0.31, −0.03], and use of the “everyone” rhetoric, *b* = 0.18, *SE* = 0.06, 95% C.I. [0.06,0.31]. In other words, social dominance beliefs were associated with colorblindness endorsement, which was associated with participants mentioning disadvantaged demographic groups less frequently and using the “colorblind inclusion” rhetoric more frequently. See Table 4 for full mediation pathway results.

Similar with our quantitative measure, we also tested the reverse pathway (see Table 5) of social dominance orientation mediating the association between colorblindness and dependent variables. Neither of the indirect effects for the reverse pathway were significant, further supporting our proposed pathway.

#### Main analyses controlling for political orientation

We also examined the mediation effect of colorblindness on the association between social dominance orientation and diversity definition shift, controlling for political orientation. The effects on quantity of disadvantaged demographic groups and non-demographic groups remained statistically significant. However, the effects on quantity of advantaged demographic groups, mention of non-specific disadvantaged groups, and use of colorblind inclusion rhetoric did not hold when controlling for political orientation. Overall, the mediation pathways held on 2 out of 5 models controlling for political orientation, suggesting that the effects only remain robust for diversity definition shift regarding including fewer disadvantaged demographic groups and more non-demographic groups in diversity.

## General discussion

Using multiple methodologies assessing White Americans’ definitions of diversity, the present research suggests that certain diversity definitions may have underlying motivations focused on maintaining the current social hierarchy in the US. In particular, White participants’ higher social dominance orientation was associated with stronger colorblind ideology endorsement, which was then associated with shifting of their definition of diversity. This shifting was associated with participants including more non-demographic groups and advantaged demographic groups in their definition, a phenomenon previously termed “broadening” diversity (i.e., including more characteristics than diversity’s original focus on protected demographic groups; Unzueta et al., 2012; Trawalter et al., 2016; Kirby et al., 2023). Participants shifted the definition further, however, by also including *fewer* disadvantaged demographic groups in their definition of diversity when they were higher in anti-egalitarian and colorblind motives. One possible way of shifting diversity definitions is to include so many characteristics (a “laundry list”) that the original focus on demographics is obscured (Dingel and Sage, 2020). However, increasing the number of characteristics while simultaneously reducing the number of protected characteristics (relative to those lower in colorblindness) is a particularly strong demonstration of the phenomenon. This hints at the possibility of a strategic shift in diversity definition that depends on participants’ motivations related to the current social hierarchy.

These associations between anti-egalitarian and colorblind motivations with definition shifts did not replicate in some of the open-ended coding variables, where participants responded about their definition of diversity. However, anti-egalitarian belief was associated with participants using the “colorblind-inclusion” rhetoric (i.e., endorsing the notion that everyone should be included in diversity) and being less likely to include disadvantaged characteristics in their definition of diversity—with both effects mediated by colorblindness beliefs. Thus, the findings are fairly consistent overall in supporting the idea that anti-egalitarian motives are associated with colorblind beliefs thus a strategic shift in diversity definition to include more characteristics beyond disadvantaged demographic groups and fewer disadvantaged demographic characteristics.

### Theoretical implications

The present research contributes to the literature on motivated construal of diversity by showing that anti-egalitarian belief is associated with colorblindness, which in turn is associated with the type of groups dominant group members tend to include in their definitions of diversity. In addition to revealing anti-egalitarian beliefs motivating participants to “broaden” their conception of diversity by including more advantaged demographic groups, and non-demographic groups, and using the colorblind inclusion rhetoric, our findings indicate a simultaneous “narrowing” of diversity to include fewer disadvantaged demographic groups. These findings suggest that anti-egalitarian motives do not simply perpetuate a “broadening” effect of diversity; they might simultaneously engender a “narrowing” effect where dominant group members downplay the importance of enhancing the treatment of historically marginalized and oppressed groups. This simultaneous “broadening” and “narrowing” of diversity definition mirrors previous research on dominant group member’s double standard on the definition of discrimination (West et al., 2022), and extends previous research on showing the flexible definitional boundary of diversity driven by anti-egalitarian belief and colorblind motives.

Another major contribution of the present research is that we directly assessed what the concept of diversity entails for dominant group members. While diversity initiatives originally served to enhance the experiences of underrepresented minorities in the society (Edelman et al., 2001), less than half (42%) of the participants in the present study mentioned specific disadvantaged demographic groups in an open-ended response asking for their definitions of diversity. Furthermore, over thirty percent of the participants displayed “colorblind inclusion” rhetoric—claiming that everyone should be included in diversity, or that no particular groups should be prioritized over others. Consistent with the findings of Dingel and Sage (2020), these patterns of White’s definitions of diversity generally reflect a colorblind approach to defining diversity.

Relatedly, the present research contributes to the existing literature on colorblind racial ideology by showing another potential downstream consequence of colorblind ideology—the strategic “broadening” and “narrowing” of diversity among dominant group members. With the increasingly pervasive endorsement of colorblindness in the society (Apfelbaum et al., 2012), it is possible that a shifted definition of diversity will also pervade over time, ultimately distracting from diversity initiatives’ original focus on disadvantaged demographic groups.

### Limitations and future directions

A key methodological limitation of the current study concerns its correlational nature, given our interest in understanding motivations for shifting definitions of diversity. We examined the association between anti-egalitarian belief, colorblindness, and diversity definitions with mediation analyses, but we cannot draw causal inferences from our data. Relatedly, our mediation model draws on cross-sectional data, which limits our ability to rule out the possibility of other models (see Fiedler et al., 2018) or establish temporal inferences based on the mediation analysis. Future research should manipulate anti-egalitarian belief or colorblindness experimentally to establish the causal effects of social hierarchy-enhancing beliefs on diversity definition shifts.

In addition, the main findings should be interpreted with caution given the possibility of political orientation and anti-egalitarian thoughts both being associated with diversity definition shift. In the present research, anti-egalitarian beliefs were highly correlated with political orientation, in line with previous research (Wilson and Sibley, 2013). When controlling for political orientation, anti-egalitarian belief’s association with diversity definition shift became less robust. When using political orientation as an alternative predictor in the mediation model, political orientation was associated with higher colorblindness beliefs, which was associated with inclusion of fewer disadvantaged demographic groups in diversity. While we cannot tease apart the effects of political orientation and anti-egalitarian belief in the current study, future research should examine the unique effect of anti-egalitarian belief on diversity definition shift.

The present research hypothesized that anti-egalitarian belief and colorblindness would be associated with targeted broadening and narrowing of diversity. However, other mechanisms related to individuals’ egalitarian beliefs (e.g., right wing authoritarianism, ingroup favoritism) could also be associated with diversity definition shifts. Additionally, our manipulation only had a significant effect on one of the dependent variables, thus the overall effect of the manipulation is not robust. Future research could use a different threat manipulation−for example, information activating more self-relevant realistic threat (Rios et al., 2018) might lead dominant group individuals to shift their definitions of diversity.

To obtain a general sense of participants’ definitions of diversity, we provided participants a variety of demographic groups and asked them to decide which groups to include across four diversity initiatives and their own definition of diversity. We recoded participants’ answers into binary variables and calculated the number of groups participants included out of the three group categories (i.e., disadvantaged demographic group, non-demographic group, advantaged demographic group). However, there might be more nuances within each category in participants’ decision-making process.

Given our interest in disadvantaged demographic groups in general (not minoritized racial groups in particular), the use of colorblindness instead of a more general identity-blind measure was somewhat mismatched with the dependent measures. Although colorblind ideologies might function similarly to identity-blind diversity ideologies, this has not been established thus far. For example, people interpret gender-blind and colorblind ideologies differently (Martin, 2023).

## Conclusion

Discourse around who should be included in diversity has gone through substantial changes over the last few decades. This study shows that dominant group members’ definitions of diversity closely align with their anti-egalitarian motives and colorblindness endorsement. A colorblind mindset may be one key motivator for White Americans to “broaden” their conception of diversity to include groups that were not the traditional focus of diversity and “narrow” their conception of diversity to include fewer oppressed or marginalized groups. Understanding the divergent definitions of diversity and the possible motivations underlying strategic shift could offer insights into the paradoxes of implementation of diversity-related policies. Taken together, these findings contribute to previous literature on motivated construal of diversity and have implications for the subtle ways in which colorblind ideology may be enacted.

## Data availability statement

The datasets presented in this study can be found in online repositories. The names of the repository/repositories and accession number(s) can be found below: https://osf.io/32ahc/.

## Ethics statement

The studies involving humans were approved by the Purdue’s Institutional Review Board. The studies were conducted in accordance with the local legislation and institutional requirements. The participants provided their written informed consent to participate in this study.

## Author contributions

JZ: Formal Analysis, Writing−original draft, Writing−review and editing. TK: Conceptualization, Methodology, Project administration, Supervision, Writing−review and editing.

## Footnotes

1 We pre-registered the study to have a 2-level design where we manipulate racial demographic change at participants’ university to examine how racial demographic change impacts participants’ definitions of diversity. As discussed in the method section, we ultimately collapsed the data across experimental conditions and shifted our focus to exploratory analyses. Thus, our predictions were secondary predictions that we did not pre-register.

2
https://osf.io/b2dgz/?view_only=f23026a9e9d34e21ada3763882d24b84

3 We also ran all the analyses controlling for condition, and condition did not have a significant effect on any analysis we ran in the paper.

4 In the alternative model, we found interactions between colorblindness and anti-egalitarian beliefs on inclusion of disadvantaged demographic groups, *F*(1, 494) = 15.34, *p* < 0.001, and inclusion of advantaged demographic groups, *F*(1, 493) = 19.46, *p* < 0.001. Specifically, at average and high levels, but not low levels, of anti-egalitarian beliefs, colorblindness was associated with participants including fewer disadvantaged demographic groups in diversity. However, at medium and low levels, but not high levels, of anti-egalitarian beliefs, colorblindness was associated with participants including more advantaged demographic groups. These unexpected findings tentatively suggest that even egalitarian-minded participants demonstrate a “broadening” pattern when they hold colorblind beliefs systems. Anti-egalitarians’ tendency to include advantaged demographic groups may reflect a desire to be included in diversity and multiculturalism, which dovetails with research suggesting that dominant groups are concerned about being excluded from diversity (Plaut et al., 2011). Because we did not theorize these divergent patterns across dependent measures *a priori* (and interactions often require large sample sizes to achieve sufficient statistical power; Blake and Gangestad, 2020), we believe that these findings should be interpreted cautiously until they are replicated.
