# Supplementary material for: Who counts as diverse? The strategic broadening and narrowing of diversity
Source: Front Psychol. 2024 Feb 6;15:1297846. doi: 10.3389/fpsyg.2024.1297846 (PMC10876864; doi:10.3389/fpsyg.2024.1297846)
Supplement: Supplementary file 1 [file Data_Sheet_1.PDF]

Who Counts as Diverse? The Strategic Broadening and Narrowing of Diversity

Supplementary Analyses

Pre-registered Analyses

Based on our pre-registration (https://osf.io/b2dgz/?view_only=f23026a9e9d34e21ada3763882d24b84), we predicted that participants who receive the information on racial demographic trends at Purdue will shift their definition of diversity to include groups that are not historically stigmatized, such as people with different learning and working styles and people from demographic groups that have not been historically marginalized. Specifically, in an independent-samples t-test, participants reading about a racial demographic shift will more strongly endorse the inclusion of non-demographic groups in diversity initiatives and definition, relative to those in the control condition.

Three independent samples t-tests were conducted to compare participants' overall desire to include the three types of groups (disadvantaged demographic groups, non-demographic groups, and advantaged demographic groups) in control and racial demographic shift conditions. There was a significant difference in the desire to include non-demographic groups $t(496) = 1.65$, $p < .001$, such that participants included fewer non-demographic groups when reading about demographic shifts compared to the control condition—this was exactly counter to hypotheses. There was no significant difference in the scores for disadvantaged demographic groups, $t(496) = 0.17$, $p = .909$, or advantaged demographic groups, $t(496) = 1.20$, $p = .275$. See Table S1 for full statistics.

Inconsistent with our hypothesis, participants reading about a racial demographic shift did not strongly endorse the inclusion of non-demographic groups in diversity initiatives and definition, compared to those in the control condition; instead, they indicated a lower desire to include non-demographic groups compared to those in the control condition. One possibility was that the demographic shift information made race and ethnicity salient, which led to the inclusion

of fewer non-demographic characteristics through demand characteristics. Alternatively, learning that there were more students of minoritized racial backgrounds at their university led participants to think that these characteristics were an important priority in initiatives, but non-demographic characteristics were not. Because another study (included in a separate manuscript: Kirby, Zeng, & McMahon, in progress) did not replicate the present effect, however, we were not convinced that this was a robust finding.

Supplementary Mediation Analysis

We tested whether our mediation model replicates on the primary measure of diversity definition in our pre-registration---mean of participants' overall desire to include disadvantaged demographic groups, non-demographic groups, and advantaged demographic groups. See Table S2 for full statistics of mediation pathway results and see Table S3 for indirect effects from the mediation models and the reverse pathways. Our proposed mediation pathway replicated for all diversity definition variables in this measure.

Exploratory Moderation Analysis

We also tested an exploratory model examining whether social dominance orientation moderated the association between colorblindness and participants' inclusion of non-demographic groups, disadvantaged demographic groups, and advantaged demographic groups in diversity definition using the PROCESS macro version 4.2 (Hayes, 2013) with 10,000 bootstrapped samples. See Table S4 for full statistics for the moderation models.

The moderation model indicated that both colorblindness and social dominance orientation were significantly and negatively associated with the quantity of disadvantaged demographic groups participants included. There was also a significant interaction between colorblindness and social dominance orientation, $F(1, 494) = 15.34$, $p < .001$. In particular,

stronger colorblindness was associated with less inclusion of disadvantaged demographic groups for participants with average, $b = -0.20$, $SE = 0.04$, $p < .001$, or high anti-egalitarian beliefs, $b = -0.32$, $SE = 0.06$, $p < .001$, but not for those low in social dominance orientation, $b = -0.08$, $SE = 0.06$, $p = .162$. These results indicate that colorblindness is only associated with participants' including fewer disadvantaged demographic groups in their diversity definition when they indicate average or strong anti-egalitarian beliefs.

The moderation model also indicated that colorblindness, but not social dominance orientation, was significantly associated with quantity of advantaged demographic groups participants included. There was also a significant interaction between colorblindness and social dominance orientation, $F(1, 493) = 19.46$, $p < .001$. In particular, colorblindness was associated with participants' inclusion of advantaged demographic groups when social dominance orientation is low, $b = 0.21$, $SE = 0.03$, $p < .001$ or medium, $b = 0.10$, $SE = 0.03$, $p < .001$, but not when social dominance orientation is high, $b = -0.01$, $SE = 0.04$, $p = .877$. These results indicate that colorblindness is only associated with participants' including more advantaged demographic groups in their diversity definition when they do not indicate strong anti-egalitarian beliefs.

The interaction term was not significant for the quantity of non-demographic groups included and for any of the content coding variables.

The findings showed that although social dominance orientation moderates the association between colorblindness and the inclusion of disadvantaged demographic groups and advantaged demographic groups in diversity definition, the interaction does not replicate on the inclusion of non-demographic groups and on all of our content coding variables. Taken together, this alternative moderation model is less robust across measures than our initial exploratory

analysis reported in the main text. However, it may be worth better understanding in follow-up research to see if the patterns replicate. For example, anti-egalitarians and egalitarians may broaden diversity in different ways because they have different strategies for enacting colorblindness.

Supplementary Mediation Analysis Controlling for Political Orientation

We also examined the mediation effect of colorblindness on the association between social dominance orientation and diversity definition shift, controlling for political orientation. See Table S5 and S6 for full mediation statistics.

Social dominance orientation was significantly associated with colorblindness, $b = 0.45$, $SE = 0.06$, $p < .001$. Colorblindness, in turn, was significantly associated with including fewer disadvantaged demographic groups, $b = -0.10$, $SE = 0.04$, $p = .016$, more non-demographic groups, $b = 0.25$, $SE = 0.10$, $p = .018$, when controlling for social dominance orientation (path *b*).

The mediation models showed significant indirect effects for disadvantaged demographic groups and non-demographic groups when controlling for political orientation. Specifically, social dominance orientation was associated with colorblind endorsement, which was associated with participants including fewer disadvantaged demographic groups, $b = -0.05$, $SE = 0.02$, 95% C.I. [-.09, -.01], and more non-demographic groups, $b = 0.11$, $SE = 0.05$, 95% C.I. [.02, .21]. The effects on quantity of advantaged demographic groups, mention of non-specific disadvantaged groups, and use of colorblind inclusion rhetoric did not hold when controlling for political orientation.

Supplementary Mediation Analysis with Political Orientation as Alternative Predictor

We tested whether political orientation is associated with colorblindness and thus with diversity definition shift. The mediation model was only statistically significant for participants'

inclusion of disadvantaged demographic groups. Specifically, political orientation is associated with colorblindness, $b = 0.71$, $SE = 0.04$, $p < .001$ (path *a*). Colorblindness, in turn, was significantly associated with including fewer disadvantaged demographic groups. The mediation models showed significant indirect effects, $b = -0.11$, $SE = 0.04$, 95% C.I. [-.19, -.04]. See table S7 and S8 for full mediation statistics.

Table S1

Independent Samples t-test Results for Diversity Definition Inclusion

	Control		Demographic Shift				
	M	*SD*	*M*	*SD*	*t*(496)	*p*	Cohen's *d*
Disadvantaged Demographic Groups	8.30	1.13	8.29	1.21	0.17	.909	1.17
Non-demographic Groups	5.80	2.46	5.41	2.86	1.65	<.001	2.67
Advantaged Demographic Groups	2.20	0.84	2.18	0.81	0.21	.275	0.82

Table S2

Mediation Pathway Results for Diversity Definition Shift

	b	*SE*	*p*
Model: SDO → Colorblindness → Desire to Include Disadvantaged Demographic Groups			
a (SDO → Colorblindness)	0.79	0.06	<.001
b (Colorblindness→ Inclusion of Groups)	-0.10	0.02	<.001
c (SDO → Inclusion of Groups)	-0.33	0.03	<.001
c' (Direct Effects)	-0.26	0.03	<.001
Model: SDO → Colorblindness → Desire to Include Non-demographic Groups			
a (SDO → Colorblindness)	0.79	0.06	<.001
b (Colorblindness → Inclusion of Groups)	0.14	0.04	<.001
c (SDO → Inclusion of Groups)	-0.05	0.05	.364
c' (Direct Effects)	-0.16	0.06	.007
Model: SDO → Colorblindness → Desire to Include Advantaged Demographic Groups			
a (SDO → Colorblindness)	0.79	0.06	<.001
b (Colorblindness → Inclusion of Groups)	0.16	0.03	<.001
c (SDO → Inclusion of Groups)	0.01	0.05	.886
c' (Direct Effects)	-0.12	0.06	.036

Table S3

Indirect Effects from Mediation Models

	Social Dominance Orientation → Colorblindness → Dependent Variable			Color Evasion → Social Dominance Orientation → Dependent Variable		
Dependent Variable	*b*	*SE*	95% CI	*b*	*SE*	95% CI
Inclusion of Disadvantaged Demographic Groups	-0.07	0.02	[-.11, -.04]	-0.08	0.01	[-.10, -.05]
Inclusion of Non-demographic Groups	0.11	0.03	[.05, .18]	-0.05	0.02	[-.08, -.01]
Inclusion of Advantaged Demographic Groups	0.12	0.03	[.07, .18]	- 0.04	0.02	[-.07, .001]

Table S4
Moderation Results for Diversity Definition Shift Variables

Dependent Variable	Factor	*b*	*SE*	*t*/*Z*	*p*	95% CI
Quantity of Disadvantaged Demographic Groups Included	CE	0.18	0.09	1.99	0.05	[.00, .37]
	SDO	0.28	0.15	1.83	0.07	[-.02, .58]
	CE*SDO	-0.14	0.04	-3.92	<.001	[-.21, -.07]
Quantity of Non-demographic Groups Included	CE	0.54	0.23	2.36	.019	[.09, .98]
	SDO	0.15	0.38	0.39	.699	[-.60, .89]
	CE*SDO	-0.10	0.09	-1.14	.256	[-.27, .07]
Quantity of Advantaged Demographic Groups Included	CE	0.39	0.07	5.73	<.001	[.26, .52]
	SDO	0.45	0.11	3.98	<.001	[.23, .67]
	CE*SDO	-0.12	0.03	-4.41	<.001	[-.17, -.06]
Mention of Specific Disadvantaged Groups	CE	-0.29	0.17	-1.68	.093	[-.63, .05]
	SDO	-0.40	0.29	-1.37	.172	[-.23, .30]
	CE*SDO	0.08	0.07	1.22	.222	[-.05, .21]
Mention of Non-specific Disadvantaged Group	CE	0.03	0.21	0.15	.884	[-.38, .45]
	SDO	0.37	0.34	1.11	.265	[-.28, 1.03]
	CE*SDO	-0.10	0.08	-1.15	.251	[-.26, .07]
Mention of Non-Demographic Groups	CE	-0.08	0.28	-0.30	.766	[-.63, .47]
	SDO	0.26	0.44	0.59	.553	[-.61, 1.13]
	CE*SDO	0.00	0.10	0.02	.986	[-.20, .20]
Mention of Advantaged Demographic Groups	CE	-0.25	0.46	-0.54	.589	[-1.14, .65]
	SDO	-0.55	0.77	-0.71	.478	[-2.05, .96]
	CE*SDO	0.07	0.19	0.35	.725	[-.30, .44]
Use of Colorblind Inclusion Rhetoric	CE	0.31	0.19	1.65	.099	[-.06, .68]
	SDO	0.07	0.32	0.22	.824	[-.56, .71]
	CE*SDO	-0.04	0.07	-0.52	.605	[-.18, .11]

Note. CE=color evasion, SDO=social dominance orientation

Table S5

Mediation Pathway Results Controlling for Political Orientation

	b	*SE*	*p*
Model: SDO → Colorblindness → Quantity of Disadvantaged Demographic Groups			
a (SDO → Colorblindness)	0.45	0.06	<.001
b (Colorblindness → Quantity of Groups)	-0.10	0.04	.016
(Political Orientation → Quantity of Groups)	-0.10	0.05	.035
c (SDO → Quantity of Groups)	-0.29	0.06	<.001
c' (Direct Effects)	-0.25	0.06	<.001
Model: SDO → Colorblindness → Quantity of Non-demographic Groups			
a (SDO → Colorblindness)	0.45	0.06	<.001
b (Colorblindness → Quantity of Groups)	0.25	0.10	.018
(Political Orientation → Quantity of Groups)	0.11	0.12	.321
c (SDO → Quantity of Groups)	-0.17	0.15	.243
c' (Direct Effects)	-0.28	0.15	.065
Model: SDO → Colorblindness → Quantity of Advantaged Demographic Groups			
a (SDO → Colorblindness)	0.45	0.06	<.001
b (Colorblindness → Quantity of Groups)	0.05	0.03	.102
(Political Orientation → Quantity of Groups)	0.14	0.04	<.001
c (SDO → Quantity of Groups)	-0.02	0.04	.575
c' (Direct Effects)	-0.05	0.05	.297
Model: SDO → Colorblindness → Mention of Specific Disadvantaged Demographic Group			
a (SDO → Color Evasion)	0.45	0.06	<.001
b (Colorblindness→ Mention of Group)	-0.04	0.08	.645
(Political Orientation → Mention of Group)	-0.14	0.09	.127
c' (Direct Effects)	-0.03	0.12	.772
Model: SDO → Colorblindness → Mention of Non-specific Disadvantaged Demographic Group			
a (SDO → Colorblindness)	0.45	0.06	<.001
b (Colorblindness → Mention of Group)	-0.08	0.09	.394
(Political Orientation → Mention of Group)	-0.26	0.11	.015
c' (Direct Effects)	0.10	0.14	.484
Model: SDO → Colorblindness → Mention of Non-demographic Group			
a (SDO → Colorblindness)	0.45	0.06	<.001
b (Colorblindness → Mention of Group)	-0.12	0.13	.340
(Political Orientation → Mention of Group)	0.10	0.14	.492

c' (Direct Effects)	0.24	0.18	.172
Model: SDO → Colorblindness → Mention of Advantaged Demographic Group			
a (SDO → Colorblindness)	0.45	0.06	<.001
b (Colorblindness → Mention of Group)	0.03	0.22	.888
(Political Orientation → Mention of Groups)	-0.29	0.25	.246
c' (Direct Effects)	-0.21	0.35	.552
Model: SDO → Colorblindness → Use of Colorblind Inclusion Rhetoric			
a (SDO → Colorblindness)	0.45	0.06	<.001
b (Colorblindness → Use of Rhetoric)	0.16	0.08	.055
(Political Orientation → Use of Rhetoric)	0.14	0.10	.147
c' (Direct Effects)	-0.12	0.12	.329

Table S6

Indirect Effects from Mediation Models Controlling for Political Orientation

Dependent Variable	*b*	*SE*	95% CI
Quantity of Disadvantaged Demographic Groups	-0.05	0.02	[-.10, -.01]
Quantity of Non-demographic Groups	0.11	0.05	[.02, .21]
Quantity of Advantaged Demographic Groups	0.02	0.02	[-.004, .05]
Mention of Specific Disadvantaged Demographic Group	-0.02	0.04	[-.09, .06]
Mention of Non-specific Disadvantaged Demographic Group	-0.04	0.05	[-.13, .05]
Mention of Non-demographic Group	-0.06	0.06	[-.19, .06]
Mention of Advantaged Demographic Group	-0.01	0.10	[-.21, .20]
Use of Colorblind Inclusion Rhetoric	0.07	0.04	[.003, .15]

Table S7

Mediation Pathway Results for Political Orientation and Diversity Definition Shift Variables

	b	*SE*	*p*
Model: Political Orientation → Colorblindness → Quantity of Disadvantaged Demographic Groups			
a (Political Orientation → Colorblindness)	0.71	0.04	<.001

b (Colorblindness → Quantity of Groups)	-0.16	0.04	<.001
c (Political Orientation → Quantity of Groups)	-0.25	0.04	<.001
c' (Direct Effects)	-0.14	0.05	.003
Model: Political Orientation → Colorblindness → Quantity of Non-demographic Groups			
a (Political Orientation → Colorblindness)	0.71	0.04	<.001
b (Colorblindness → Quantity of Groups)	0.19	0.10	.060
c (Political Orientation → Quantity of Groups)	0.20	0.09	.027
c' (Direct Effects)	0.07	0.12	.541
Model: Political Orientation → Colorblindness → Quantity of Advantaged Demographic Groups			
a (Political Orientation → Colorblindness)	0.71	0.04	<.001
b (Colorblindness → Quantity of Groups)	0.04	0.03	.167
c (Political Orientation → Quantity of Groups)	0.16	0.03	<.001
c' (Direct Effects)	0.14	0.03	<.001
Model: Political Orientation → Colorblindness → Mention of Specific Disadvantaged Demographic Group			
a (Political Orientation → Color Evasion)	0.71	0.04	<.001
b (Colorblindness→ Mention of Group)	-0.04	0.08	.563
c' (Direct Effects)	-0.14	0.09	.104
Model: Political Orientation → Colorblindness → Mention of Non-specific Disadvantaged Demographic Group			
a (Political Orientation → Colorblindness)	0.71	0.04	<.001
b (Colorblindness → Mention of Group)	-0.06	0.09	.502
c' (Direct Effects)	-0.24	0.10	.019
Model: Political Orientation → Colorblindness → Mention of Non-demographic Group			
a (Political Orientation → Colorblindness)	0.71	0.04	<.001
b (Colorblindness → Mention of Group)	-0.07	0.12	.580
c' (Direct Effects)	0.14	0.14	.326
Model: Political Orientation → Colorblindness → Mention of Advantaged Demographic Group			
a (Political Orientation → Colorblindness)	0.71	0.04	<.001
b (Colorblindness → Mention of Group)	-0.01	0.21	.970
c' (Direct Effects)	-0.33	0.24	.176
Model: Political Orientation → Colorblindness → Use of Colorblind Inclusion Rhetoric			
a (Political Orientation → Colorblindness)	0.71	0.04	<.001
b (Colorblindness → Use of Rhetoric)	0.14	0.08	.089

c' (Direct Effects)	0.12	0.09	.201

Table S8

Indirect Effects from Mediation Models with Political Orientation as Predictor

Dependent Variable	*b*	*SE*	95% CI
Quantity of Disadvantaged Demographic Groups	-0.11	0.04	[-.19, -.04]
Quantity of Non-demographic Groups	0.13	0.07	[-.004, .27]
Quantity of Advantaged Demographic Groups	0.03	0.02	[-.01, .07]
Mention of Specific Disadvantaged Demographic Group	-0.03	0.06	[-.14, .07]
Mention of Non-specific Disadvantaged Demographic Group	-0.04	0.06	[-.17, .08]
Mention of Non-demographic Group	-0.05	0.09	[-.22, .12]
Mention of Advantaged Demographic Group	-0.01	0.15	[-.31, .27]
Use of Colorblind Inclusion Rhetoric	0.10	0.06	[-.01, .21]
